# Analysis of diverse β-lactamases presenting high-level resistance in association with OmpK35 and OmpK36 porins in ESBL-producing *Klebsiella pneumoniae*

**DOI:** 10.1016/j.sjbs.2022.02.036

**Published:** 2022-02-25

**Authors:** Hasan Ejaz

**Affiliations:** Department of Clinical Laboratory Sciences, College of Applied Medical Sciences, Jouf University, Al Jouf 72388, Saudi Arabia

**Keywords:** *Klebsiella pneumoniae*, Porins, OmpK35, OmpK36, Drug resistance, ESBL, β-lactamases

## Abstract

Emerging extensively drug-resistant (XDR) *Klebsiella pneumoniae* due to the production of β-lactamases and porin loss is a substantial worldwide concern. This study aimed to elucidate the role of outer membrane porin (OMP) loss, AmpC, and carbapenemases among extended-spectrum β-lactamase (ESBL)-producing *K. pneumoniae* strains with XDR phenotype. This study analyzed 79 *K. pneumoniae* from several clinical sources and detected ESBLs in 29 strains co-harbored with other β-lactamases using standard microbiological practices and phenotypic procedures. Minimum inhibitory concentrations (MICs) were determined against several antibiotics using Microscan WalkAway plus. OMP analysis was carried out using sodium dodecyl sulfate–polyacrylamide gel electrophoresis. ESBL, AmpC, and carbapenemase genes were detected using molecular methods. The microbiological analysis discovered 29 (36.7%) ESBL strains of *K. pneumoniae*, which showed the co-existence of 7 (24.1%) AmpC β-lactamases and 22 (75.9%) carbapenemases. Porin loss of OmpK35 was observed in 13 (44.8%) and OmpK36 in 8 (27.5%) *K. pneumoniae* strains. The strains were significantly associated with the intensive care unit (ICU) (*p* = 0.006) and urinary sources (*p* = 0.004). The most commonly detected gene variants in each β-lactamase class included 16 (55.2%) *bla*_CTX-M−1_, 7 (100%) *bla*_CYM-2_, 11 (50%) *bla*_NDM-1_, and integron-1 was detected in 21/29 (72.4%) strains. MICs of cephalosporin, fluoroquinolone, carbapenem, aminoglycoside, and β-lactam combinations demonstrated a high number of XDR strains. Tigecycline (2 µg/mL MIC_50_ and >32 µg/mL MIC_90_) and colistin (1 µg/mL MIC_50_ and 8 µg/mL MIC_90_) presented lower resistance. ESBL *K. pneumoniae* strains with OmpK35 and OmpK36 porin loss demonstrate conglomerate resistance mechanisms with AmpC and carbapenemases, leading to emerging XDR and pan drug resistance.

## 1 Introduction

*Klebsiella pneumoniae* has emerged as one of the most challenging multidrug-resistant (MDR) organisms, defying the last line of drugs and rapidly becoming untreatable. This organism shows a significant tendency to acquire drug-resistant traits and can cause a range of infections, including pneumonia and bloodstream infections (Palmieri et al., 2020). *K. pneumoniae* is widely regarded as an opportunistic pathogen and colonizes healthy individuals' skin, throat, and intestinal tract (Podschun and Ullmann, 1998). A diverse number of clones widely distributed geographically have been identified, causing a large number of infections. *K. pneumoniae* has recently evolved as a significant public health threat due to a large increase in the rate of nosocomial infections ascribed to MDR strains that harbor extended-spectrum β-lactamases (ESBLs) and carbapenemases (Wyres et al., 2020). *K. pneumoniae* and other Gram-negative pathogens are becoming increasingly carbapenem-resistant (CR), a global concern that might undermine the usefulness of a fundamentally essential antibiotic class used to treat life-threatening infections (Rice, 2008).

According to the World Health Organization, *K. pneumoniae* has become increasingly recognized as a priority antimicrobial-resistant (AMR) pathogen that demands new management strategies (WHO, 2017). The initial genetic source of resistance is the expression of carbapenemases encoded on enormous plasmids (Mathers et al., 2015). Hydrolysis of carbapenems by these enzymes renders them ineffective. The plasmids are transported vertically from the parent cell to the daughter cells during cell division or horizontally through conjugal transfer. Antibiotic influx across the outer membrane of CR *K. pneumoniae* is limited by chromosomal alteration of the main outer membrane porins, OmpK35 and OmpK36 (Pulzova et al., 2017). *K. pneumoniae* now comprises AMR phenotypes ranging from CR to colistin-resistant, classifying it as a highly resistant pathogen (Bi et al., 2017).

Outer membrane porins (OMPs) typically comprise trimers, which serve as water-filled protein channels for the transportation of hydrophilic substances through the external membrane. Porins are also a vector for phages and bacteriocins and play a major structural role in protecting cell integrity with peptidoglycan and lipopolysaccharide (Achouak et al., 2001). Antibiotics gain access to OMPs to reach the periplasm. The porin channels appear to be the critical entry point for β-lactams, which are typically hydrophilic and charged (Li et al., 2015). In *Escherichia coli*, two key porins, OmpC and OmpF, have been thoroughly characterized. In comparison to OmpC, OmpF has a slightly bigger functional pore; as a result, molecules pass through the pore of OmpF with more ease than with OmpC (Nikaido and Rosenberg, 1983, Nikaidoet al., 1983). OmpK35 and OmpK36, two main porins of *K. pneumoniae*, are identical to OmpF and OmpC, respectively. Only OmpK36 is expressed by the majority of ESBL-producing *K. pneumoniae* strains, whereas most non-ESBL-producing *K. pneumoniae* contain both OmpK35 and OmpK36 (Hernández-Allés et al., 1999). The lack of OmpK35 has been observed in ESBL producers, while OmpK36 loss is most often found in CR *K. pneumoniae* (Palasubramaniamet al., 2009, Skurniket al., 2010).

The group of β-lactam drugs is the foundation of the antibiotic armamentarium because they inhibit bacterial cell wall synthesis. Because β-lactam drugs infiltrate through porins into the exterior membrane of different Gram-negative bacterial strains, porin loss or deficiency governs antibiotic resistance (Nikaido, 1989). The *K. pneumoniae* outer membrane represents an obstacle to permeability that modulates a drug’s capacity to affect its intracellular target. *K. pneumoniae* is rapidly becoming untreatable using last-line antibiotics. This study focused on the role of OMPs among ESBL-producing *K. pneumoniae* strains, their drug resistance spectrum, and the molecular diversity of different β-lactamase genes.

## 2 Materials and methods

### 2.1 Ethical considerations

The study followed the ethics guidelines of the World Medical Association (WMA) Declaration of Helsinki and did not include any human or animal subjects (WMA, 2013). The Institutional Research Ethics Committee approved the study project via approval no. 08-04-42/Expedited.

### 2.2 Specimen collection

The clinical specimens were collected from the most prominent specialist hospital of Al Jouf, Saudi Arabia. Diverse clinical specimens, such as blood, urine, tracheal, CSF, pus swabs, secretions, endotracheal tubes (ETTs), and wound swabs were processed to isolate *K. pneumoniae*. The cephalosporin-resistant strains of *K. pneumoniae* were initially included in the study based on minimum inhibitory concentrations (MICs) until confirmed by molecular techniques.

### 2.3 *K. Pneumoniae* isolation

The clinical specimens were cultured on blood, chocolate, and MacConkey media except for urine processed on cystine lactose electrolyte deficient media (Nosheen et al., 2021). The identification of the bacteria was accomplished using Gram’s stain, morphology, oxidase, indole, citrate, sugar fermentation, and Microscan WalkAway Plus (Beckman Coulter, USA). A total of 79 *K. pneumoniae* strains obtained from inpatients and outpatients were isolated from the clinical specimens.

### 2.4 Phenotypic detection of ESBLs

The detection of ESBLs in *K. pneumoniae* was the inclusion criteria and was performed by earlier-described phenotypic methods (Ejaz et al., 2013). Quality control (QC) of the tests was performed using ATCC *E. coli* 25,922 and *K. pneumoniae* 700,603 strains. Non-ESBL-producing *K. pneumoniae* were excluded from further processing.

### 2.5 AmpC detection

AmpC β-lactamase enzyme production was observed with the help of cefoxitin discs by making 0.5 McFarland suspensions and inoculating them onto the surface of MH agar plates incubated overnight at 37 °C (Younas et al., 2018). The representative strains were inoculated onto MH agar, and 30 µg cefoxitin discs alone and with boronic acid were also transferred onto the plates. The cefoxitin and boronic acid zones were compared to cefoxitin alone, and an increase of ≥5 mm in the zone showed AmpC production. Non-toxin-producing ATCC *E. coli* 25922 was used as a negative QC in the test.

### 2.6 Carbapenemase production

The cloverleaf formation assessed carbapenemase enzyme production in the modified Hodge test (MHT). The ATCC *E. coli* (25922) suspension prepared compared to 0.5 McFarland’s standard was diluted ten folds and streaked on MH agar. The test and QC *K. pneumoniae* ATCC strains (BAA-1705) were inoculated on the plate, and cloverleaf indentations characterized the tested isolates as carbapenemase producers (Ejaz et al., 2020).

### 2.7 Minimum inhibitory concentrations (MICs)

Phenotypically characterized ESBL-producing *K. pneumoniae* were processed for MIC determination against cephalosporin, fluoroquinolones, carbapenems, aminoglycosides, β-lactam combinations, and several other antibiotic classes using Microscan WalkAway plus. The broth microdilution technique was applied wherever necessary to ensure the accuracy of the MICs (Jang et al., 2013). MIC breakpoints were observed and interpreted to report the resistance profile of *K. pneumoniae* according to the Clinical Laboratory Standards Institute (CLSI, 2020).

### 2.8 Detection of OMPs

OMP isolation was performed using the method described by Hernandez-Alle (Hernández-Allés et al., 1999). Mueller Hinton broth was used to grow fresh *K. pneumoniae* strains. *K. pneumoniae* cell envelopes were separated by centrifugation at 100,000*g* for 1 h at 4 °C. By using sodium lauryl sarcosinate insoluble material, the OMPs were removed from the solution. Samples were further boiled for 5 min for electrophoresis analysis. Then, OMP electrophoretic analysis was carried out by using the sodium dodecyl sulfate–polyacrylamide gel electrophoresis (SDS-PAGE) method, which was performed with 11% acrylamide (11%), bisacrylamide (0.35%), and SDS (0.1%) in the presence of Laemmli’s buffer. Coomassie blue stain was used for the staining of bands.

### 2.9 Molecular characterization of β-lactamase genes and integrons

Polymerase chain reaction (PCR) was utilized to report the different AMR genes. The singleplex and multiplex techniques were utilized to optimize the PCR protocol for ESBL genes *bla*_TEM_, *bla*_SHV_, and *bla*_CTX-M_, and the AmpC gene *bla*_CMY-2._ For the detection of carbapenem genes *bla*_NDM,_ *bla*_IMP,_ and *bla*_OXA-48,_ the protocol described earlier, and PCR amplification using primers and cycling parameters were adopted (Poirelet al., 2004, Poirelet al., 2011, Ejazet al., 2021). Multiplex PCR methodology was used to amplify the *Int-1*, –*2,* and –*3* (Machado et al., 2005). ESBL genes *bla*_TEM_, *bla*_SHV_, and *bla*_CTX-M_ were sequenced to analyze the amino acid substitutions at the active site and analyzed using BlastN, BlastP (NCBI), and ExPASy (SIB Group).

### 2.10 Statistical analysis

GraphPad Prism 6.0 and IBM SPSS v.26 were used for statistical analyses. Descriptive statistics were performed to compute the frequencies and percentages, and the chi-square test was used to calculate p-values. P-values of less than 0.05 were considered significant in this work.

## 3 Results

### 3.1 Isolation of *K. Pneumoniae* strains

**Fig. 1:**
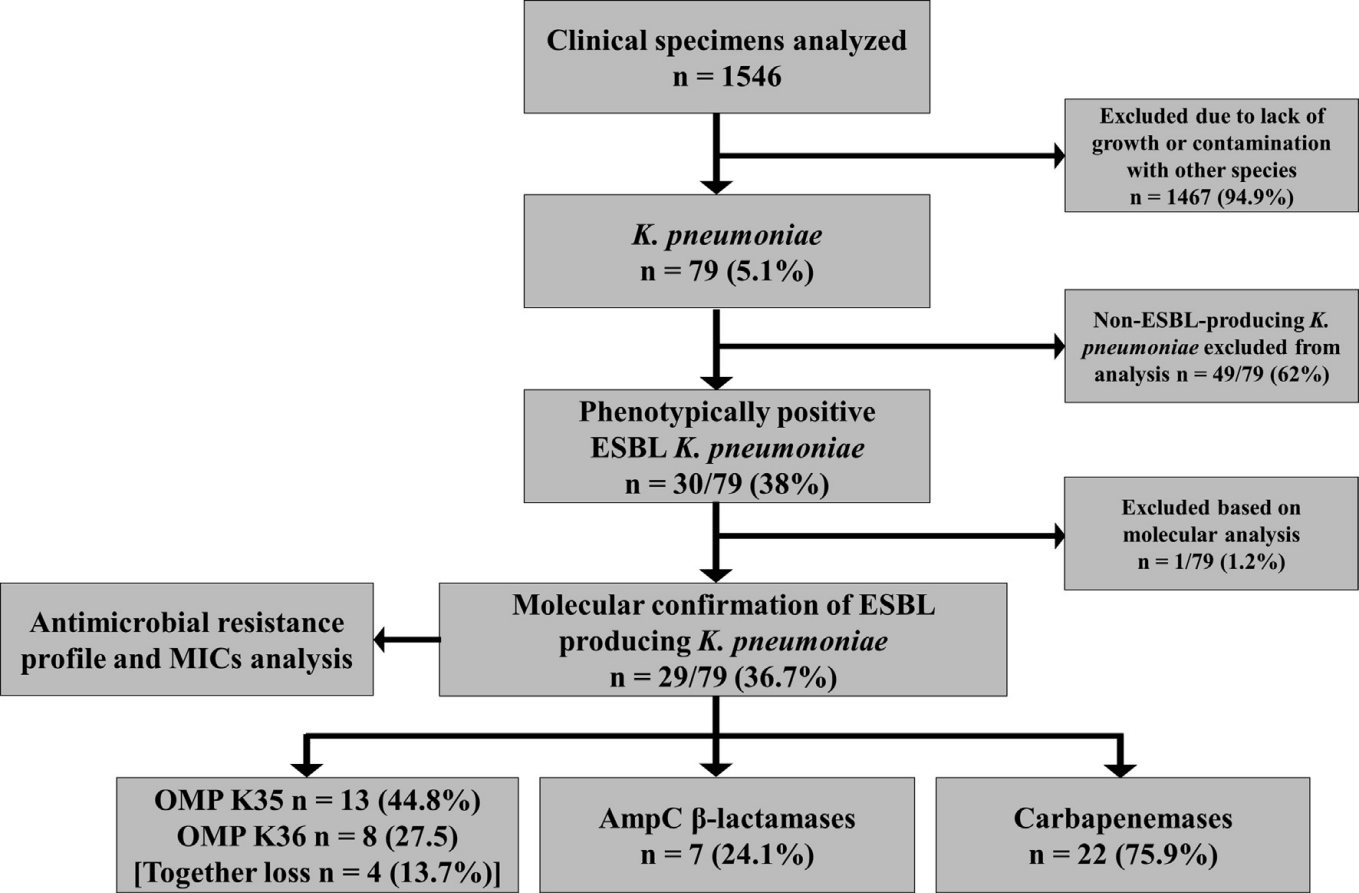
Isolation and processing of clinical strains.

Of 1546 disparate clinical sources, 79 *K. pneumoniae* strains were included. The reminders strains were excluded due to lack of growth or contamination with other species. *K. pneumoniae* strains were processed for phenotypic identification of ESBLs, which presented 30/79 (38%) ESBL positive isolates. The molecular analysis confirmed 29/79 (36.7%) ESBL producers, which further showed the co-existence of 7 (24.1%) AmpC β-lactamases and 22 (75.9%) carbapenemases. Loss of the OmpK35 porin was observed in 13 (44.8%) strains and OmpK36 in 8 (27.5%) strains, whereas 4 isolates showed the loss of both OmpK35 and OmpK36 simultaneously (Fig. 1).

### 3.2 Demographic characteristics

No statistically significant (*p* = 0.69) difference was observed among the ESBL-producing and –non-producing *K. pneumoniae* based on sex. There was no significant association of the patients with hospital wards except for the intensive care unit (ICU), which showed *p* = 0.006. ESBL producers were predominantly detected among 11/29 (37.9) ICU, 8/29 (27.6%) medical, and 4/29 (13.8%) coronary care unit (CCU) patients. *K. pneumoniae* demonstrated a significant association (*p* = 0.004) with the urinary sources. In contrast, no statistically significant relationship was observed among the other clinical sources. The infected patients included in the study had a median age of 45.41 ± 16.31 years (Table 1).

**Table 1:** Characteristics of patients infected with *K. pneumoniae*.

Characteristics		ESBL (n = 29) n (%)	Non-ESBL (n = 50) n (%)	p-value
Sex	Male	17 (58.6)	27 (54)	0.69
	Female	12 (41.4)	23 (46)	
Wards	CCU	4 (13.8)	2 (4)	0.11
	ICU	11 (37.9)	6 (12)	0.006
	Medical	8 (27.6)	11 (22)	0.57
	Pediatric	2 (6.9)	9 (18)	0.16
	Isolation	2 (6.9)	4 (8)	0.85
	OPD	1 (3.4)	11 (22)	0.02
	Surgery	1 (3.4)	7 (14)	0.13
Sources	Urine	14 (48.3)	9 (18)	0.004
	Tissue	3 (10.3)	6 (12)	0.83
	Wound swab	3 (10.3)	10 (20)	0.26
	Blood	4 (13.8)	6 (12)	0.81
	CVC Tip	1 (3.4)	5 (10)	0.28
	Sputum	4 (13.8)	14 (28)	0.14
Age		45.41 ± 16.31	47.42 ± 11.21	0.51

### 3.3 *K. Pneumoniae* β-lactamases from the clinical sources

**Fig. 2:**
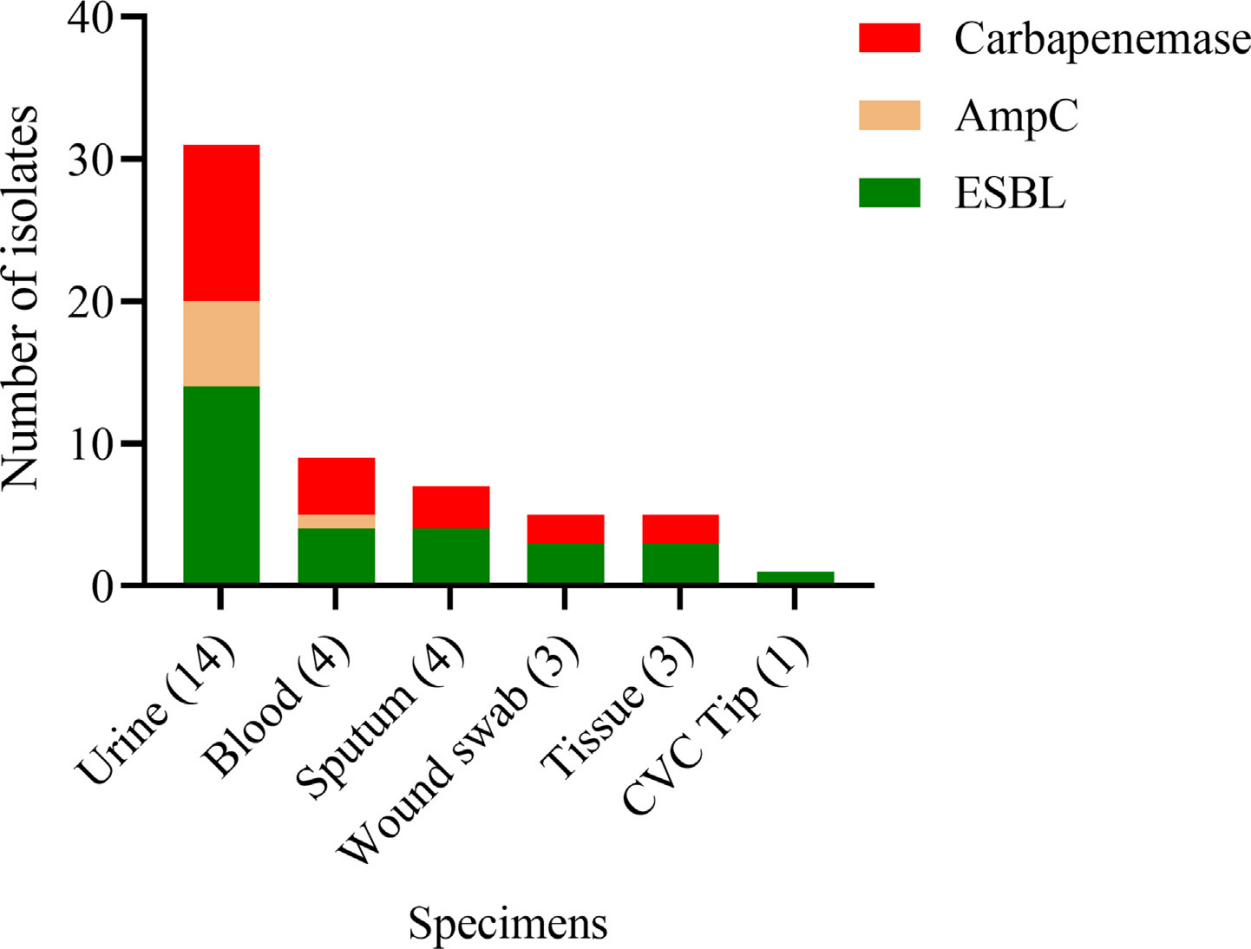
Distribution of β-lactamases in *K. pneumoniae* isolated from clinical sources (n = 29).

**Fig. 3:**
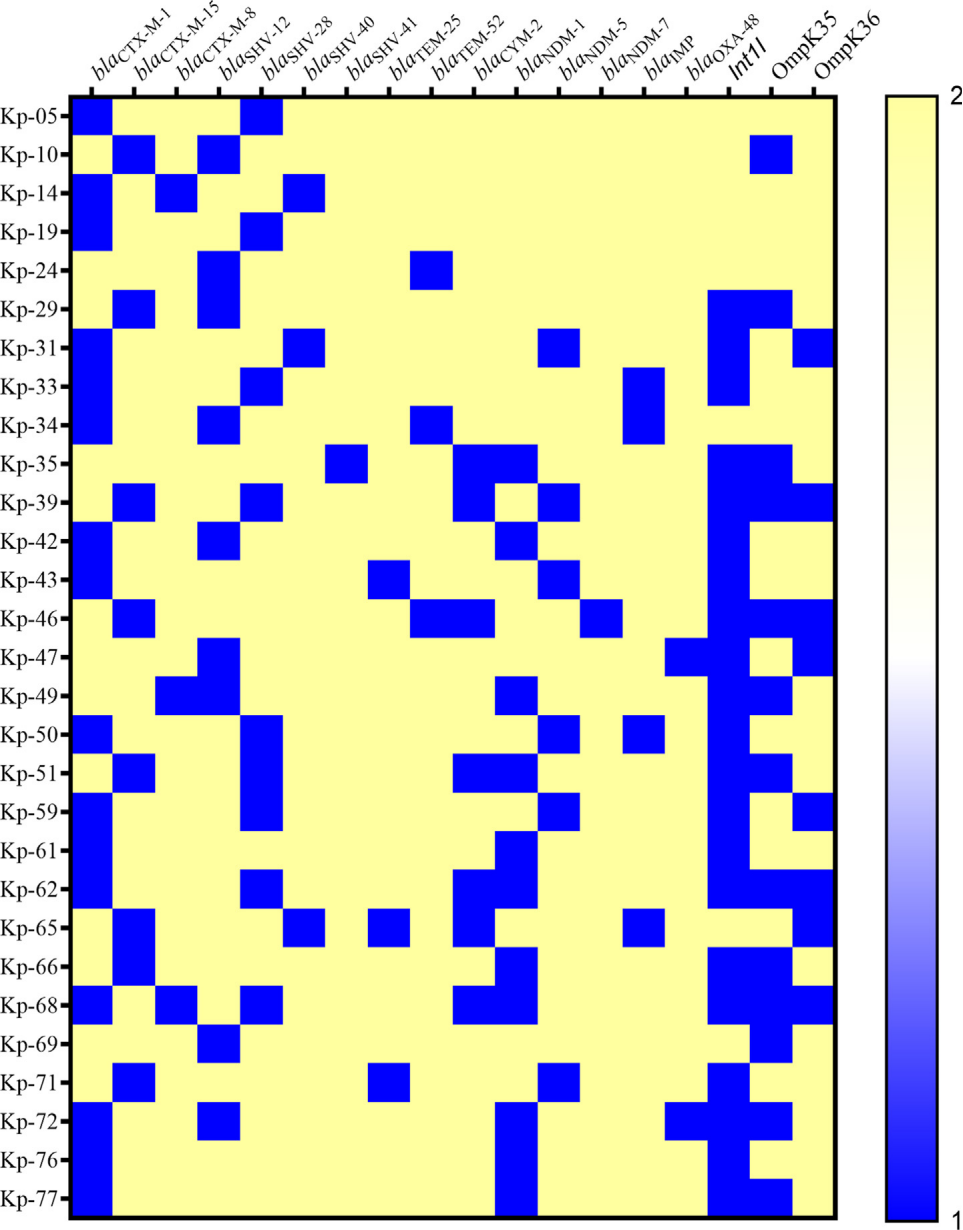
**Heat map x-axis shows the frequency of ESBLs, AmpC, carbapenemases, and integron genes.** OMPK 35 and OMPK 36 porin loss can be observed in several isolates. Each *K. pneumoniae* clinical strain is shown on the y-axis. The scale’s blue color given a numeric value of 1 represents the gene’s occurrence and porin loss in each isolate to indicate high-level resistance due to these mechanisms. The yellow color with a numeric value of 2 represents the absence of a particular gene and the presence of porins in *K. pneumoniae* strains. The co-existence of several genes can be observed in each *K. pneumoniae* strain.

Several ESBL-producing *K. pneumoniae* from the clinical sources were found to co-harbor AmpC and carbapenemase enzymes. The highest number of AmpC (6; 42.9%) and carbapenemase (11; 78.6%) enzymes were observed among the urinary isolates, as shown in Fig. 2. The predominantly detected ESBL variants were 16 (55.2%) *bla*_CTX-M−1_, 9 (31%) *bla*_SHV-12_, 9 (31%) *bla*_SHV-28_, and 8 (27.6%) *bla*_CTX-M−15_. All of the 7 detected AmpC β-lactamases were of the *bla*_CYM-2_ type. Among the carbapenemases, the most frequent variants were 11 (50%) *bla*_NDM-1_, 6 (27.3%) *bla*_NDM-5_, and 4 (18.2%) *bla*_IMP_ (Table 2). The heat map in Fig. 3 shows various genes of ESBLs, AmpC, carbapenemases in each of the *K. pneumoniae*. Twenty-one (72.4%) *K. pneumoniae* strains exhibited the presence of the integron-1 (*Int-1*) gene. The co-existence of drug-resistant genes and porin loss of OmpK35 (13/29) and OmpK35 (8/29) is presented for each *K. pneumoniae* strain. Porin loss was seen in all AmpC producers, while at least one or both porins were lost in 10 of the carbapenemase-producing *K. pneumoniae*.

**Table 2:** Distribution of β-lactamase gene variants among *K. pneumoniae* (n = 29).

β-lactamase Type	*bla* variants	Number	Percentage
ESBLs (n = 29)	*bla*_CTX-M-1_	16	55.2
	*bla*_SHV-12_	9	31
	*bla*_SHV-28_	9	31
	*bla*_CTX-M-15_	8	27.6
	*bla*_CTX-M-8_	3	10.3
	*bla*_SHV-40_	3	10.3
	*bla*_TEM-25_	3	10.3
	*bla*_TEM-52_	3	10.3
	*bla*_SHV-41_	1	3.4
AmpC β-lactamases (n = 7)	*bla*_CYM-2_	7	100
Carbapenemases (n = 22)	*bla*_NDM-1_	11	50
	*bla*_NDM-5_	6	27.3
	*bla*_IMP_	4	18.2
	*bla*_OXA-48_	2	9.1
	*bla*_NDM-7_	1	4.5

### 3.4 Antibacterial resistance, MIC_50,_ and MIC_90_ of different antibiotics

*K. pneumoniae* strains included in the study showed extensive drug resistance (XDR) against several antibiotic classes, and all of them were resistant to cefotaxime, and cefuroxime. High bacterial resistance was observed against 28/29 (96.6%) ampicillin-sulbactam and cefepime, 27/29 (93.1%) co-trimoxazole, 26/29 (89.7%) moxifloxacin, ceftazidime and ertapenem, 25/29 (86.2%) piperacillin-tazobactam and ciprofloxacin, 24/29 (82.8%) co-amoxiclav, aztreonam, cefoxitin, imipenem, levofloxacin, meropenem, and 22/29 (75.9%) tobramycin. There were 14/29 (48.3%) organisms that showed moderate resistance to amikacin with 32 µg/mL MIC_50_ and >512 µg/mL MIC_90_. The only choice of antibiotics among these XDR strains was tigecycline and colistin, which manifested resistance to 9/29 (31%) and 6/29 (20.7%) strains, respectively. The MIC of tigecycline was 2 µg/mL MIC_50_ and >32 µg/mL MIC_90_, while colistin presented values of 1 µg/mL MIC_50_ and 8 µg/mL MIC_90_ as shown in Table 3.

**Table 3:** *In vitro* antibacterial resistance, MIC_50,_ and MIC_90_ of clinical *K. pneumoniae* strains (n = 29).

**Antibiotics**	**Breakpoint** µg/mL	**MIC_50_** µg/mL	**MIC_90_** µg/mL	**Resistant strains** **n (%)**
Amikacin	≥ 64	32	> 512	14 (48.3)
Co-amoxiclav	≥ 32/16	≥ 128/64	≥ 128/64	24 (82.8)
Ampicillin-sulbactam	≥ 32/16	≥ 256/128	≥ 256/128	28 (96.6)
Aztreonam	≥ 16	> 16	> 16	24 (82.8)
Cefepime	≥ 16	> 128	> 128	28 (96.6)
Cefotaxime	≥ 4	> 32	> 32	29 (1 0 0)
Cefoxitin	≥ 32	> 128	> 128	24 (82.8)
Ceftazidime	≥ 16	> 128	> 128	26 (89.7)
Cefuroxime	≥ 4	> 256	> 256	29 (1 0 0)
Ciprofloxacin	≥ 1	> 32	> 32	25 (86.2)
Ertapenem	≥ 2	> 32	> 32	26 (89.7)
Gentamicin	≥ 16	64	> 128	20 (69)
Imipenem	≥ 4	> 32	> 32	24 (82.8)
Levofloxacin	≥ 2	> 64	> 64	24 (82.8)
Meropenem	≥ 4	> 32	> 32	24 (82.8)
Moxifloxacin	> 1	> 64	> 64	26 (89.7)
Piperacillin-tazobactam	≥ 128/4	> 128/4	> 128/4	25 (86.2)
Tigecycline	≥ 2	2	> 32	9 (31)
Tobramycin	≥ 16	32	> 128	22 (75.9)
Co-trimoxazole	≥ 4/76	16/304	> 32/608	27 (93.1)
Colistin	≥ 4	1	8	6 (20.7)

MIC minimum inhibitory concentration.

## 4 Discussion

The wide prevalence and distribution of highly resistant clones of *K. pneumoniae* have become a paramount global health disquietude. *K. pneumoniae* imposes significant pressure on the healthcare system due to the emergence of XDR strains, leading to severe nosocomial infections. The efficacy of available drugs has been rapidly reduced and has evoked a search for efficient new drugs (Padilla et al., 2010). Beta-lactam antimicrobial agents form the foundation of our antibiotic armamentarium. The enzymatic deactivation of antibiotics and reduction of membrane permeability are some of the commonly identified primary mechanisms of β-lactam resistance (Nikaido and Pagès, 2012). The *K. pneumoniae* outer membrane has two non-specific porins, OmpK35 and OmpK36, which play a vital role in transporting the most frequently used antibiotics. XDR due to the production of diverse β-lactamases is a crucial concern and jeopardizes the therapeutic approaches when associated with porin loss, leading to pan drug resistance.

The current study reports an overall 36.7 % of ESBL producers of *K. pneumoniae,* which was higher than reported in Pakistan (30.1%) (Ejaz et al., 2017), India (23.6%) (Ananthan and Subha, 2005), and less than found in Iran (57.5%) (Hashemi et al., 2014) and China (83.3%) (Shi et al., 2002). A comparatively lower prevalence was noted in the Western Pacific (24.6%) and Europe (22.6%) (Gonlugur et al., 2004), potentially due to specific factors such as sample size and collection technique disparities, including analytical method variations. Most obtained strains were from patients admitted to ICU, similar to a Malaysian hospital study (Lau et al., 2021). Many *K. pneumoniae* recovered from urine specimens agreed with an earlier report (Hashemi et al., 2014). In the current report, OmpK35 porin loss was observed in 13 strains of ESBL and OmpK36 in 8 strains. Six carbapenemase-producing strains presented OmpK35 and 7 OmpK36 porin loss, while three of these *K. pneumoniae* exhibited loss of both porins together. The absence of porins in conjunction with ESBL production is demonstrated in CR *K. pneumoniae* (Martínez-Martínez et al., 1999). The underlying resistance mechanisms include point mutations that can result in protein changes, premature truncation, or insertion sequences resulting in porin gene interruption.

Regarding molecular characterization, the most commonly found ESBL variants were (55.2%) *bla*_CTX-M−1_, (27.6%) *bla*_CTX-M−15_, followed by 3 (10.3%) *bla*_CTX-M−8_, with *bla*_SHV-12_, *bla*_SHV-28_ (31%), *bla*_TEM-25_ and *bla*_TEM-52_ (10.3%). The *bla*_CTX-M−15_ (27.6%) rate in this study is somehow similar to the study from Lebanon (Arabaghian et al., 2019). The *bla*_CTX-M_ has been reported worldwide as the most common ESBL type, and in most local areas, it can easily exceed the proportion of *bla*_SHV_ and *bla*_TEM_ ESBLs (Jorgensen et al., 2010). Hashemi *et al*. reported 62.5% *bla*_CTX-M−15_ of 48 ESBL-producing *K. pneumoniae*, consistent with the current findings (Hashemi et al., 2014). Another report from Brazil corroborated this study because they also reported the highest rate of *bla*_CTX-M_ in *K. pneumoniae* (Carvalhaes et al., 2010). Ferreira et al. worked on 25 ESBL producing *K. pneumoniae* obtained from a Brazilian ICU and found *bla*_TEM_ in100% of the isolates (Ferreiraet al., 2018).

The expression of major porins may be altered by genetic mutations, which result in either decreased porin expression or a complete loss of porin (Martínez-Martínez et al., 1999). Porin loss is a major source of resistance to several antimicrobials, most notably β-lactams. All the strains were further analyzed for the occurrence of the AmpC enzyme, carbapenemase production, and porin expression. Surprisingly, many co-harboring carbapenemase (82.7%) and AmpC (24.1%) producers were also detected in this work. The porin loss among *K. pneumoniae* in the current study was 44.8% for OmpK35, 27.5% OmpK36, and 13.7% for both porins. An analysis from Hong Kong showed similar findings, with 33.8% of isolates demonstrating OmpK36 loss and 11.3% both porins loss (Ho et al., 2016). The results also agree with prior studies finding that porin loss in ESBL-producing *K. pneumoniae* could be liable for carbapenem resistance (Skurniket al., 2010, Ferreiraet al., 2018). In conjunction with porin loss and drug efflux, carriage of ESBL or AmpC has been identified as contributing factors to carbapenem resistance in *Enterobacteriaceae* (Ferreiraet al., 2018). The analysis of different resistant gene determinants revealed CTX M type ESBLs, AmpC, and porin loss to be highly prevalent among carbapenem-resistant isolates, which corroborates the results observed by Ho *et al.* in Hong Kong (Ho et al., 2016). Nordmann and Mammeri found that CTX-M-15 and CMY-2 are responsible for reducing the sensitivity to several antibiotics (Nordmann and Mammeri, 2007). Most of the *K. pneumoniae* co-harboring CYM-2 gene and porin loss presented a high degree of multidrug resistance, including carbapenems (Yang et al., 2009).

NDM is the most commonly distributed metallo-β-lactamase enzyme identified in *Enterobacteriaceae* (Hsu et al., 2017). Carbapenem-resistant *K. pneumoniae* may harbor more than one gene, which is not surprising because *bla*_NDM_ carrying plasmids are also associated with *bla*_OXA-48_ and *bla*_VIM_ (Nordmann et al., 2011). Two *bla*_NDM-1_ isolates were found with both porins loss and were highly resistant to the available drugs. These strains were co-harboring *bla*_CYM-2_ genes, which suggested the loss of porin and AmpC production, reducing carbapenem susceptibility, as noted by Martínez-Martínez (Martínez-Martínez et al., 1999).

The detection of two *bla*_OXA-48_ -harboring *K. pneumoniae* strains is in good accordance with an Iranian study (Hashemi et al., 2014). Alterations in the porin synthesis and acquisition of specific enzymes hydrolyze several antibiotics, leading to XDR strains. The MIC_50_ and MIC_90_ showed several bacterial strains that were highly resistant to the different groups of drugs. The lowest resistance rates were observed against colistin and tigecycline, agreeing with a prior study from Brazil (Vivan et al., 2017). However, emerging colistin resistance has been reported from China (Liu et al., 2021). An extensive spectrum of drug resistance has been observed in OmpK35- and OmpK36-deficient *K. pneumoniae*, in association with ESBL and AmpC production, leading to the development of substantial resistance against the β-lactam antibiotics, fluoroquinolone, and aminoglycosides (Hamzaoui et al., 2018). *K. pneumoniae* OmpK35 and OmpK36 loss results in a higher rate of antibacterial resistance and, in particular, leads to increased resistance to carbapenems and other drugs. This study’s limitations were that the sequencing of the porin genes could not be performed to determine the point mutations, deletions, and insertions.

## 5 Conclusion

The findings of this study present a disquieting situation of XDR *K. pneumoniae* that have acquired resistance to the last therapeutic options to treat life-threatening infections. OmpK35 and OmpK36 porin loss associated with carbapenemases and AmpC β-lactamases in ESBL-producing *K. pneumoniae* make these strains particularly recalcitrant to almost all of the present-day repertoire of antibiotics. Carbapenem resistance is not only limited to carbapenemase production but is also linked to porin loss. The expansion and dissemination of such clones in healthcare settings may pose serious therapeutic problems in the future, leaving tigecycline and colistin as the only current therapeutic options.

## Funding

The author’s work was supported through grant number “375213500” from the Deputyship for Research and Innovation, Ministry of Education in Saudi Arabia.

## Declaration of Competing Interest

The authors declare that they have no known competing financial interests or personal relationships that could have appeared to influence the work reported in this paper.
